# Long-Term Delivery of an Anti-SIV Monoclonal Antibody With AAV

**DOI:** 10.3389/fimmu.2020.00449

**Published:** 2020-03-17

**Authors:** José M. Martinez-Navio, Sebastian P. Fuchs, Desiree E. Mendes, Eva G. Rakasz, Guangping Gao, Jeffrey D. Lifson, Ronald C. Desrosiers

**Affiliations:** ^1^Department of Pathology, Miller School of Medicine, University of Miami, Miami, FL, United States; ^2^Wisconsin National Primate Research Center, University of Wisconsin, Madison, WI, United States; ^3^Gene Therapy Center, University of Massachusetts Medical School, Worcester, MA, United States; ^4^AIDS and Cancer Virus Program, Frederick National Laboratory for Cancer Research, Frederick, MD, United States

**Keywords:** gene therapy, AAV vector, long-term expression, broadly neutralizing antibodies, HIV/SIV cure, immunotherapy, prophylaxis, rhesus monkeys

## Abstract

Long-term delivery of anti-HIV monoclonal antibodies using adeno-associated virus (AAV) holds promise for the prevention and treatment of HIV infection. We previously reported that after receiving a single administration of AAV vector coding for anti-SIV antibody 5L7, monkey 84-05 achieved high levels of AAV-delivered 5L7 IgG1 *in vivo* which conferred sterile protection against six successive, escalating dose, intravenous challenges with highly infectious, highly pathogenic SIVmac239, including a final challenge with 10 animal infectious doses (1). Here we report that monkey 84-05 has successfully maintained 240–350 μg/ml of anti-SIV antibody 5L7 for over 6 years. Approximately 2% of the circulating IgG in this monkey is this one monoclonal antibody. This monkey generated little or no anti-drug antibodies (ADA) to the AAV-delivered antibody for the duration of the study. Due to the nature of the high-dose challenge used and in order to rule out a potential low-level infection not detected by regular viral loads, we have used ultrasensitive techniques to detect cell-associated viral DNA and RNA in PBMCs from this animal. In addition, we have tested serum from 84-05 by ELISA against overlapping peptides spanning the whole envelope sequence for SIVmac239 (PepScan) and against recombinant p27 and gp41 proteins. No reactivity has been detected in the ELISAs indicating the absence of naturally arising anti-SIV antibodies; moreover, the ultrasensitive cell-associated viral tests yielded no positive reaction. We conclude that macaque 84-05 was effectively protected and remained uninfected. Our data show that durable, continuous antibody expression can be achieved after one single administration of AAV and support the potential for lifelong protection against HIV from a single vector administration.

## Introduction

Gene therapy has come of age. Almost 50 years after its inception, gene therapy is now considered a promising treatment option for several human diseases including cancer, genetic disorders and infectious disease (2). Gene therapies can work by several mechanisms: replacing defective genes with healthy ones, adding new genes to help the body fight or treat disease, or deactivating problematic genes (3). Importantly, for any of these gene therapy approaches, achieving long-term delivery of the transgene remains a key, infrequently realized goal. Recombinant adeno-associated virus (AAV) vectors have been widely used for such gene delivery applications because of their safety and cost-efficiency: a single injection can result in long-term expression of the transgene product (4). Also, recombinant AAV is ideal as a delivery vehicle in some additional respects: the only protein expressed from it comes from the inserted transgene; it can effectively transduce terminally differentiated non-dividing cells; and it shows little or no integration into host genome sequences (5–10).

One potentially important application of the AAV system is the delivery of broadly neutralizing antibodies as a gene therapy approach against HIV (1, 11–14). For that purpose, recombinant AAVs encoding neutralizing antibodies are inoculated into the host and the antibody or antibodies of interest will then be directly expressed from the transduced cells. Thus, the immune system is bypassed in the sense that no immune response to an immunogen or vaccine is needed; the desired final products (broadly neutralizing antibodies) are delivered directly to the host. This approach against HIV has been made realistically possible in recent years thanks to the isolation and characterization of a remarkable collection of potent, broadly-neutralizing, monoclonal antibodies from select individuals (15–17). These antibodies have been extensively characterized in the laboratory and some have moved to clinical trials, where they have shown activity (18–22). They have the potential to prevent infection and also serve as a therapeutic approach complementing or even substituting antiretroviral drugs (11, 14, 18, 20–25). Importantly, the use of AAV voids the need for repeated administrations of purified antibody to maintain therapeutic levels in circulation. Due to its simplicity and ease of deployment, the approach is ideal for global use (26).

One main problem has been encountered in the applicability of this approach. Host antibodies generated against the delivered antibody (generally known as anti-drug antibodies or ADAs) can reduce its functionality and concentration thus drastically reducing its effectiveness (1, 11, 24, 27–32). The large repertoire of endogenously generated antibodies present in any individual has gone through a complex checks and balances system to be allowed into circulation (33). The antibodies being made in one individual are different from the antibodies being made in another individual (34). In addition, the potent broadly-neutralizing anti-HIV antibodies that one would want to use for these applications are typically highly evolved over a prolonged period of time. Due to years of affinity maturation, they exhibit unusually high levels of somatic hypermutation in the variable domains and many have accumulated unusual structural features (35, 36) which are generally required for potent neutralization and breadth (37). This high level of mutation likely enhances the immunogenicity of these antibodies when delivered to a host other than the one in which those particular sequences originated. Interestingly, when characterizing the humoral responses to the AAV-delivered antibodies we and others have found that the variable regions were predominantly or exclusively targeted (1, 11, 28, 30). We have also reported a highly significant correlation of the magnitude of the host anti-antibody response with the distance from germline of the AAV-delivered antibody: the more mutated, the more immunogenic (28). ADAs are in fact a common problem with many gene therapy approaches (38, 39). However, if the hurdle of the ADAs can be overcome, the promise of the AAV-delivery of antibodies against HIV could be realized (12). Here, we report that monkey 84-05 has maintained 240–350 μg/ml of anti-SIV antibody 5L7 for over 6 years in the absence of detectable ADAs. Our data show that durable, continuous antibody expression can be achieved after a single administration of AAV and support the potential for lifelong protection against HIV from a single vector administration.

## Materials and Methods

### Macaque 84-05

The animal used in this study is a Mamu B^*^08-neg B^*^17-neg female Indian-origin rhesus macaque (*Macaca mulatta*), originally housed at the New England Primate Research Center in a biosafety level 3 animal containment facility in accordance with the standards of the Association for Assessment and Accreditation of Laboratory Animal Care and the Harvard Medical School Animal Care and Use Committee. Research was conducted according to the principles described in the *Guide for the Care and Use of Laboratory Animals* and was approved by the Harvard Medical School Animal Care and Use Committee (40). Macaque 84-05 tested negative for the presence of antibodies to HIV and AAV1 capsid prior to AAV administration. At the time this manuscript was written, the monkey was 14 years old and weighted 6.4 kg. She was administered AAV encoding for 5L7 antibody when she was 7 years old and weighted 5.5 kg (1). At week 108 (about 2 years) post-AAV administration, the monkey was transferred to and subsequently housed at the Wisconsin National Primate Research Center and cared for in accordance with the guidelines of the Weatherall Report under a protocol approved by the University of Wisconsin Graduate School Animal Care and Use Committee.

### Recombinant AAV

Coding sequences were designed *in silico*, codon-optimized and gene-synthesized (GenScript). 5L7 immunoadhesin sequences (11) served as a template and full-length antibodies were constructed by adding CH1 domain and CL domain of rhesus IgG to the already known immunoadhesin sequences. 5L7 sequences originate from recombinant anti-SIV Fab sequences (347-23h) derived from the bone marrow of SIV-infected rhesus monkeys (41). The rhesus IgG1 sequence used is based on accession no. AAF14058 and AAQ57555. Rhesus kappa light chain was designed using the constant light domain sequence from AAD02577. Synthesized fragments were then cloned into the NotI site of our AAV vector plasmids (1, 42). Production of recombinant AAVs was conducted as described previously (43). In short, HEK-293 cells were transfected with a select AAV vector plasmid and two helper plasmids to allow generation of infectious AAV particles. After harvesting transfected cells and cell culture supernatant, AAV was purified by three sequential CsCl centrifugation steps. Vector genome number was assessed by Real-Time PCR, and the purity of the preparation was verified by electron microscopy and silver-stained SDS-PAGE. The AAV genomes were encapsidated with the AAV1 serotype. Monkey 84-05 received recombinant AAV1 encoding the anti-SIV antibody 5L7, using a total dose of 1.6 × 10^13^ particles (2.9 × 10^12^ AAV vector genomes per kilogram of body weight). AAV administration was conducted by a one-time inoculation of four deep intramuscular injections (two separate 0.5 ml injections into both quadriceps).

### Recombinant 5L7 Antibody

5L7 recognizes gp120 and gp140 forms of the SIVmac239 envelope glycoprotein (28) and binds conformational epitopes involving the V3-V4 region (41). HEK-293T cells were expanded and then transfected with recombinant AAV vector plasmid coding for 5L7 antibody. Cells were washed after 4 h with pre-warmed PBS and then transferred to serum-free medium (Invitrogen). Afterwards, the antibody-containing medium was harvested, precleared by centrifugation, and filtered through a 0.22 μm-pore-size membrane. Then, IgG was affinity-purified using protein A Sepharose 4 Fast Flow (GE Healthcare), concentrated and desalted, followed by protein quantification with a Nanodrop UV spectrometer (Thermo Fisher Scientific). Antibody purity was confirmed by sodium dodecyl sulfate-polyacrylamide gel electrophoresis (SDS-PAGE) and subsequent Coomassie blue staining (Thermo Fisher Scientific).

### *In vivo* 5L7 Antibody Quantification and Anti-Drug Antibody (ADA) Responses

AAV-delivered 5L7 antibody was quantitated by standard ELISA using plate bound SIVmac239 gp140 (Immune Tech) to capture the antibody from pre-diluted serum samples and then HRP-conjugated secondary anti-rhesus IgG (Southern Biotech) as the detection method. Absorbance at 450 nm was compared to a serial dilution of purified 5L7 produced in HEK 293T cells, and the amount of antibody in serum was interpolated based on the standard curve. To measure host humoral responses to the AAV-delivered 5L7 antibody, purified recombinant 5L7 was used to coat plates. Then, serum samples from monkey 84-05 were tested at a 1:20 dilution and ADA responses were detected by means of an anti-lambda HRP-conjugated secondary antibody (Southern Biotech) in a regular ELISA (28). This secondary did not cross-react with 5L7 coated on the plates since 5L7 bears a kappa light chain. This allowed us to readily detect those anti-antibodies with a lambda light chain, which have been reported to reflect humoral responses in our previous studies (1, 28). Levels of AAV-delivered 5L7, and the corresponding ADAs, were measured for a total of 340 weeks.

### PepScan

PepScan or ELISA against a panel of 218 peptides overlapping the entire SIVmac239 envelope protein was used to detect antibody responses to the viral spike. Fifteen-mer-length peptides, each successive peptide overlapped by 11 amino acids, were obtained from the NIH AIDS Research and Reference Reagent Program. Peptides were properly resuspended and used to coat ELISA plates at 40 μg/ml in phosphate-buffered saline (PBS). Plates were then washed, blocked and incubated for 1 h at 37°C with a 1:20 dilution of the corresponding monkey sera or 5L7 antibody diluted to 2 μg/ml. Binding antibodies were detected with an HRP-conjugated goat anti-human IgG antibody (SouthernBiotech) diluted 1:1,000 in 5% non-fat powdered milk in PBS and developed with soluble tetramethylbenzidine (TMB) reagent (Calbiochem, Gibbstown, NJ). The reaction was stopped by the addition of 50 μl of acidic stop solution (SouthernBiotech, Birmingham, AL), and the optical density at 450 nm was measured using a Wallac Victor plate reader (Perkin-Elmer, Waltham, MA).

### Antibody Responses to p27 and gp41

Antibody responses against p27 and gp41 were quantitated by regular ELISA using SIVmac239 p27 purified recombinant protein (Catalog# 13446; obtained through the NIH AIDS Reagent Program, Division of AIDS, NIAID, NIH) and recombinant SIV gp41 (Catalog# 5019; ImmunoDX) to coat plates at 10 μg/ml in PBS. Plates were then washed, blocked and incubated for 1 h at 37°C with a 1:20 dilution of the corresponding monkey sera or 5L7 antibody diluted to 2 μg/ml. Antibodies binding the p27 antigen or the gp41 were detected with a goat anti-monkey secondary antibody (Catalog# 2015-05, Santa Cruz) and developed with TMB High Sensitivity Substrate Solution (Catalog# 421501, Biolegend). The reaction was stopped by the addition of 50 μl of acidic stop solution (SouthernBiotech, Birmingham, AL), and the optical density at 450 nm was measured using a Wallac Victor plate reader (Perkin-Elmer, Waltham, MA).

### Viral Load Monitoring

Plasma SIV RNA was measured using a gag-targeted quantitative real-time/digital PCR method with 6 replicate reactions analyzed per extracted sample for an assay threshold of 30 SIV RNA copies/ml (44). Cell-associated SIV RNA and DNA were measured by quantitative hybrid real-time/digital RT-PCR and PCR assays (45, 46).

## Results

### Over 6 Years of Continuous Antibody Expression *in vivo*

**Figure 1:**
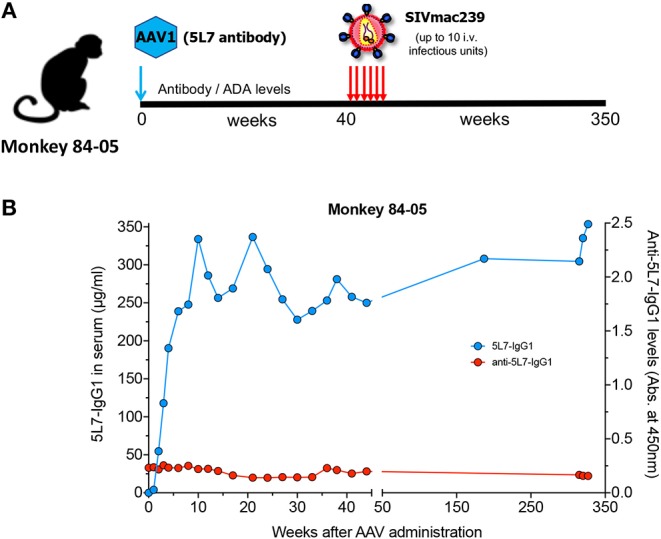
5L7 antibody levels and the corresponding anti-antibodies (ADAs) in serum following AAV administration. **(A)** Schematic of the experiment in which rhesus monkey 84-05 received intramuscular administration of AAV1 coding for antibody 5L7 and was subsequently challenged with SIVmac239 (six times: 4 times with a 1 animal infectious doses followed by a 2x dose challenge and a final 10x dose challenge). **(B)** 5L7 antibody levels (blue; plotted on left axis) and anti-5L7 antibody levels (red; plotted on right axis) in serum from monkey 84-05, quantified by ELISA.

Monkey 84-05 received a single administration of AAV on September 6th, 2012. As we reported at that time (1), starting at week 44 post-AAV inoculation and then every 3 weeks, this animal was repeatedly challenged with highly pathogenic, highly infectious SIVmac239 for a total of six intravenous administrations (see schematics in Figure 1A). The first four challenges were performed with 1 animal infectious dose (AID), followed by challenge with 2 AID and then a final 10 AID challenge. Viral load measurements in plasma from 84-05 were negative (<30 copies/ml), indicating the animal successfully resisted all six challenges (1). The antibody levels achieved remarkable values of ~270 μg/ml (1). We now report long-term follow-up on levels of 5L7 antibody and the corresponding ADAs in circulation. As shown in Figure 1B, macaque 84-05 has successfully maintained 240–350 μg/ml of anti-SIV antibody 5L7 for over 340 weeks (>6 years). Moreover, this animal has shown little or no ADA responses to the AAV-delivered 5L7 antibody (Figure 1B).

### Negative Envelope PepScan

**Figure 2:**
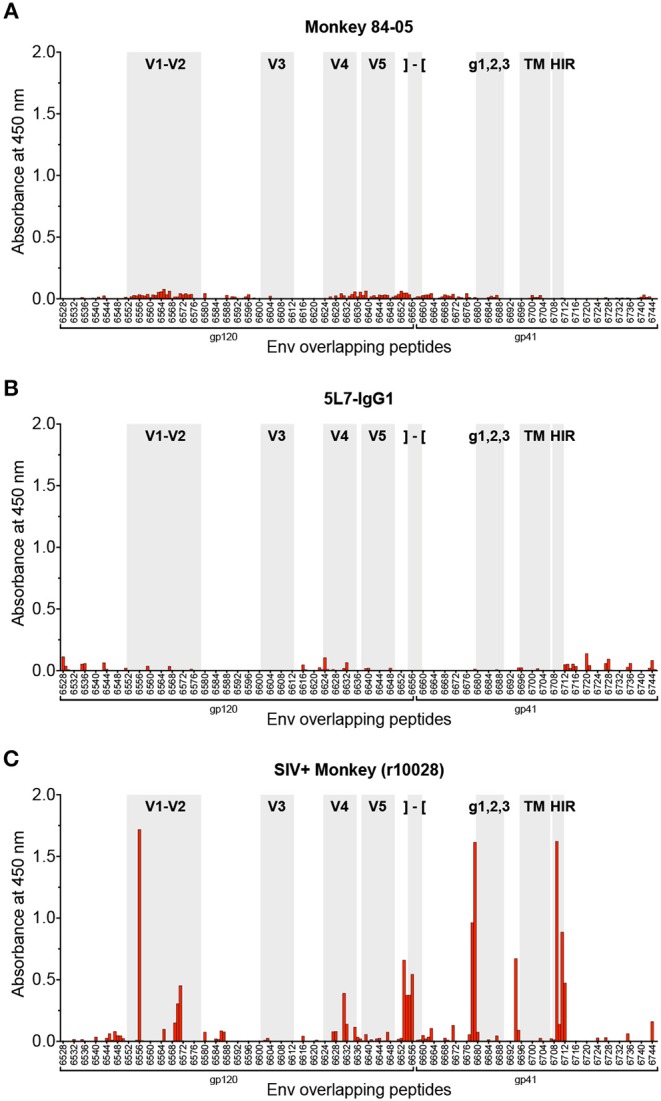
Analysis of antibody responses by PepScan. PepScan (ELISA against overlapping peptides) spanning the whole envelope sequence for SIVmac239, performed with **(A)** serum from 84-05 (week 328 post-AAV) at a 1:20 dilution; **(B)** recombinant purified 5L7 antibody at a concentration of 2 μg/ml; and **(C)** serum from a rhesus monkey experimentally infected with SIVmac239 (week 12 post-SIV infection) at a 1:20 dilution. Note: regions of interest are shadowed in gray and indicated as follows: variable regions 1–2, 3, 4, and 5 are labeled V1–V2, V3, V4, and V5, respectively; the cleavage site and beginning of gp41 are represented by brackets; g123 indicates the location of the N-linked carbohydrate sites found in gp41; TM is the transmembrane domain of gp41; and HIR stands for highly immunogenic region. Data from SIV+ monkey in panel **(C)** is representative of many such SIV+ monkeys tested previously (47, 48).

Due to the nature of the high-dose challenge employed, and the high levels of anti-envelope reactivity found in serum of this animal, one could speculate about a potential low-level infection not detected by regular viral load measurements, which could lead to development of endogenous anti-Env responses, resulting in an overestimation of the concentration of 5L7 antibody in the anti-SIVmac239 gp140 ELISA employed (see Materials and Methods). In order to rule out this potential scenario, we tested serum from 84-05 by PepScan (ELISA against overlapping peptides spanning the whole envelope sequence for SIVmac239) (47, 48). One of the most sensitive measures of infection is seroconversion. Established immunodominant regions of the envelope, i.e., the variable loops V1–V2 and V3, the C terminus of gp120, some peptides in the ectodomain of gp41, and the highly immunogenic region (HIR) at the beginning of the cytoplasmic tail of gp41(47–49) are the regions frequently targeted by antibodies. As shown in Figure 2A, the serum from this animal did not detectably react to any of the peptides in the SIVmac239 envelope PepScan. Importantly, and as expected, recombinant purified antibody 5L7 (which is known to bind a conformational epitope involving the V3-V4 region of the envelope (11, 41) and should therefore not bind the linear epitopes present in the PepScan), did not detectably bind any of the tested peptides (Figure 2B). Conversely, when positive control serum from a SIV-infected macaque (r10028) was tested on a PepScan in the same conditions, strong reactivity was shown to the immunodominant regions cited above (Figure 2C).

### No Detectable Antibody Responses to p27 or gp41 by ELISA

**Figure 3:**
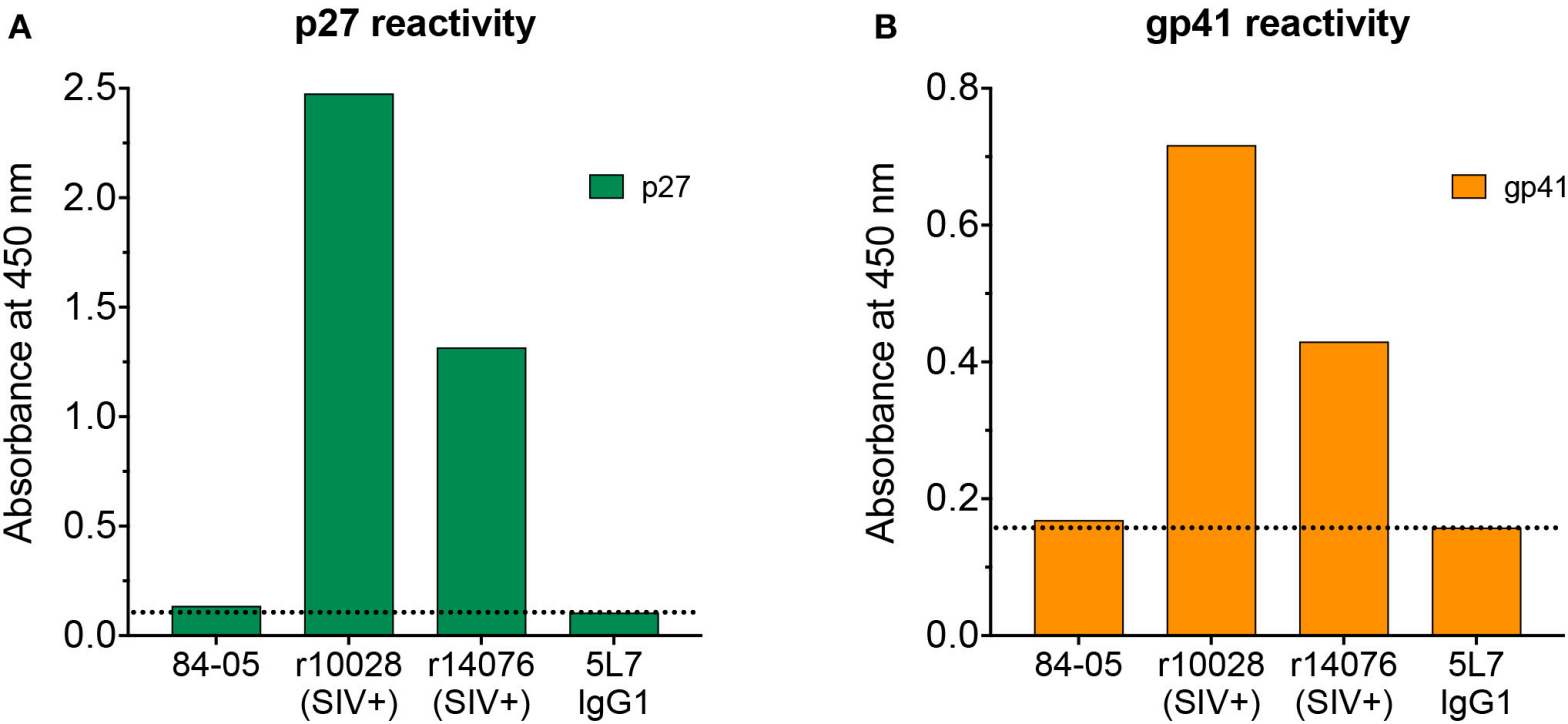
Analysis of antibody responses to p27 and gp41. Serum from 84-05 (week 328 post-AAV) at a 1:20 dilution was tested by ELISA against recombinant purified proteins p27 **(A)** gp41 and **(B)** sera from two rhesus monkeys experimentally infected with SIVmac239 (week 12 post-SIV infection) at a 1:20 dilution were used as positive controls, and recombinant purified 5L7 as a negative.

With the high levels of 5L7 antibody consistently found in serum of 84-05, meaning high reactivity to gp120, assessing a potential low-level infection can be complicated since that serum would definitely yield a positive result when tested against whole virus or recombinant gp120 by ELISA, the benchmark assays. An alternate method to test potential seroconversion is by measuring ELISA reactivity to p27 and gp41 recombinant proteins. Antibodies to both p27 and gp41 are readily detectable shortly after SIV infection and importantly, the 5L7 antibody present in the serum would not react to these proteins. Tests of serum from 84-05 (week 328 post-AAV) did not reveal any reactivity to p27 (Figure 3A), while positive control sera from two rhesus macaques experimentally infected with SIVmac239 for 12 weeks (r10028 and r14076) showed high reactivity under the same conditions. Similarly, serum from 84-05 did not detectably react against gp41, but sera from the two SIV-positive monkeys did (Figure 3B). Purified 5L7 antibody tested in parallel did not significantly bind to either p27 or gp41 (Figure 3).

### Absence of Cell-Associated Viral DNA or RNA

To further rule out a potential low-level infection that could not be detected by the aforementioned ELISA-based tests, we used ultrasensitive techniques to detect cell-associated viral DNA and RNA in PBMCs from 84-05. We did not detect SIV gag RNA or DNA in the analysis of a combined total of 1.17 × 10^8^ cell equivalents obtained over a 2-week period (week 340 and week 342; Table 1) with a nominal sensitivity of 1 copy per reaction.

**Table 1:** Ultrasensitive detection of cell-associated viral DNA and RNA in PBMCs.

	**Week 340^a^**	**Week 342^b^**
Cell associated viral DNA	<1 DNA copies/10^6^ cells	<1 DNA copies/10^6^ cells
Cell associated viral RNA	<1 RNA copies/10^6^ cells	<1 RNA copies/10^6^ cells

a A total of 7.4 × 10^7^ cells were analyzed for this time point and

b *a total of 4.3 × 10^7^ cells were analyzed for this time point. Both with a nominal sensitivity of 1 copy per reaction*.

## Discussion

Here we describe continuous, prolonged, high level delivery of an antibody to a rhesus monkey using AAV vector over 6 years of longitudinal measurements. Approximately 2% of the IgG in monkey 84-05 is derived from the vector that we inoculated more than 6 years previous. This result is consistent with the belief that muscle cells essentially do not turn over (50) and thus represent a potentially long term stable source of AAV-delivered antibody. It seems likely that monkey 84-05 will continue to produce similar levels of this antibody for the rest of its life. Importantly, this animal withstood six successive challenges with SIVmac239, including a final challenge with 10 animal infectious doses (1). By different means we show here that monkey 84-05 was effectively protected and remained uninfected: these include testing seroconversion in three different ways and the use of ultrasensitive techniques to detect cell-associated viral DNA and RNA. Due to the difficulties associated with proving curative and/or protective interventions (51), additional tests were considered such as *in vivo* CD8+ T-cell depletion and attempts at adoptive transfer of infection to naïve rhesus macaques. However, we did not want to put this precious monkey at any risk with the CD8 depletion; and, surprisingly, adoptive transfer may not be as sensitive as one would think (14).

The absence of an ADA response is almost certainly a key factor in the continuous production of the transgene product in monkey 84-05. Other examples of AAV delivery of protein to monkeys over a prolonged period have been documented in the reports of Rivera et al. (52) and Guilbaud et al. (53). Both of these reports used periodic induction of expression of an erythropoietin identical in sequence to the monkey's own erythropoietin over 5 or more years of study. Additional examples include the persistent expression of dopamine-synthesizing enzymes in the putamen reported in one monkey for 15 years in a primate model of Parkinson's disease (54); the sustained expression of human α-L-iduronidase, an important enzyme required for the lysosomal degradation of glycosaminoglycans, reported for almost 4 years after intrathecal cervical AAV9 gene delivery in four one-month-old rhesus monkeys (55); the sustained expression of alpha-1 antitrypsin for over 5 years after one AAV vector administration in alpha-1 antitrypsin deficient patients (56); and the successful expression for 3.5 years obtained in two dogs of a dystrophin gene in a canine model for human Duchenne muscular dystrophy using AAV6 and a brief course of immunosuppressants (57), or in a similar study for over 2 years in two dogs using AAV8 in the absence of immunosuppression (58). Remarkably our animal 84-05 never received any immunosuppressant. The hemophilia gene therapy arena also has good examples of long-term delivery with AAV for up to a decade of Factor IX in hemophilic humans (59) and of Factor VIII in hemophilic dogs (60). For more examples on long-term delivery with AAV of hemophilia factors, please see the following references (61–68). The long-term delivery described in our report here is significant as the first such report for very long-term delivery of an antibody, particularly given the serious difficulties that have been encountered when AAV has been used to deliver antibodies that are significantly diverged from germ line or contain unusual features (1, 14, 28, 30, 31). The findings give hope that long-term delivery of therapeutic antibodies via AAV can be consistently achieved if the ADA problem can be overcome.

What may be responsible for the absence of ADAs in 84-05 and the continued high level of production of the transgene product over this prolonged period? Factors that have been shown to influence whether, or not, ADAs are observed following AAV-mediated expression of a transgene product include the following: the particular sequence of the transgene product (69); whether the recipient is already making a similar or identical protein (39); the serotype of AAV used (38); and targeted delivery or targeted expression at particular sites or in particular tissues or cells (54, 70–76). AAV-delivered 5L7 antibody certainly has the potential to be immunogenic in rhesus monkeys since 50% of monkeys receiving it have had robust ADA responses (1). Might there be particular genetic determinants that restrict an ADA response to the 5L7 antibody in some monkeys but not others? Might the antibody repertoire present in an individual at any one time influence to what extent the 5L7 antibody may be recognized as foreign? Might 5L7 just be on the cusp of self/non-self recognition? These questions remain unanswered at least for the time being.

Like the Miami monkey (14, 77, 78), monkey 84-05 stands out as a shining example of what is possible in the realm of AAV delivery of monoclonal antibodies in the fight against HIV. The Miami monkey has maintained high levels of two anti-HIV monoclonal antibodies and complete virologic suppression of SHIV infection for more than 4 years of follow-up without any repeated administrations and without any other antiviral therapies. It is likely that the ADA problem with AAV-delivered antibodies will need to be overcome for this approach to become a consistent reality in the context of human HIV infection. A recently-published human trial of AAV to deliver the human anti-HIV monoclonal antibody PG9 revealed readily detectable ADAs in 10 of 13 recipients in the four highest dose groups and zero measurable delivery of the PG9 antibody (31, 79). We feel that monkey modeling will need to play a key role in the development of successful strategies. Our group is currently focused on simple, easy-to-apply strategies for creating tolerance in monkeys to AAV-delivered monoclonal antibodies. If satisfactory delivery methods are found, it becomes possible to envision long-term control of viral replication in the absence of antiretroviral treatment by delivering a combination of antibodies in people, and long-lasting protection when this approach is used in a prophylactic setting. The long-term expression reported here highlights the potential of AAV-mediated antibody expression for impacting HIV-1 infections worldwide.

## Data Availability Statement

All datasets generated for this study are included in the article/supplementary material.

## Ethics Statement

The animal study was reviewed and approved by The Harvard Medical School Animal Care and Use Committee and The University of Wisconsin Graduate School Animal Care and Use Committee.

## Author Contributions

The study was conceived and designed and the manuscript was composed by JM-N, SF, and RD. The experiments were performed by JM-N, SF, DM, ER, GG, and JL. Reagents that were used in the study were generated by JM-N, SF, DM, ER, GG, and JL. Data analysis was performed by JM-N, SF, DM, ER, GG, JL, and RD.

### Conflict of Interest

The authors declare that the research was conducted in the absence of any commercial or financial relationships that could be construed as a potential conflict of interest.
